# Lysyl oxidase-like 4 promotes the invasiveness of triple-negative breast cancer cells by orchestrating the invasive machinery formed by annexin A2 and S100A11 on the cell surface

**DOI:** 10.3389/fonc.2024.1371342

**Published:** 2024-03-26

**Authors:** Tetta Takahashi, Nahoko Tomonobu, Rie Kinoshita, Ken-ichi Yamamoto, Hitoshi Murata, Ni Luh Gede Yoni Komalasari, Youyi Chen, Fan Jiang, Yuma Gohara, Toshiki Ochi, I Made Winarsa Ruma, I Wayan Sumardika, Jin Zhou, Tomoko Honjo, Yoshihiko Sakaguchi, Akira Yamauchi, Futoshi Kuribayashi, Eisaku Kondo, Yusuke Inoue, Junichiro Futami, Shinichi Toyooka, Yoshito Zamami, Masakiyo Sakaguchi

**Affiliations:** ^1^ Department of Cell Biology, Okayama University Graduate School of Medicine, Dentistry and Pharmaceutical Sciences, Okayama, Japan; ^2^ Department of Pharmacy, Okayama University Hospital, Okayama, Japan; ^3^ Faculty of Medicine, Udayana University, Denpasar, Bali, Indonesia; ^4^ Department of Breast Surgery, The First Affiliated Hospital, Zhejiang University School of Medicine, Hangzhou, China; ^5^ Department of Neurology, Okayama University Graduate School of Medicine, Dentistry and Pharmaceutical Sciences, Okayama, Japan; ^6^ Medical Oncology Department of Gastrointestinal Tumors, Liaoning Cancer Hospital & Institute, Cancer Hospital of the Dalian University of Technology, Shenyang, Liaoning, China; ^7^ Department of Interdisciplinary Science and Engineering in Health Systems, Okayama University, Okayama, Japan; ^8^ Department of Microbiology, Tokushima Bunri University, Sagamihara, Japan; ^9^ Department of Biochemistry, Kawasaki Medical School, Okayama, Japan; ^10^ Division of Tumor Pathology, Near InfraRed Photo-Immuno-Therapy Research Institute, Kansai Medical University, Osaka, Japan; ^11^ Faculty of Science and Technology, Division of Molecular Science, Gunma University, Kiryu, Japan; ^12^ Department of General Thoracic Surgery and Breast and Endocrinological Surgery, Okayama University Graduate School of Medicine, Dentistry and Pharmaceutical Sciences, Okayama, Japan

**Keywords:** breast cancer, lysyl oxidase, annexin A2, S100A11, plasmin, cancer microenvironment

## Abstract

**Background:**

Our earlier research revealed that the secreted lysyl oxidase-like 4 (LOXL4) that is highly elevated in triple-negative breast cancer (TNBC) acts as a catalyst to lock annexin A2 on the cell membrane surface, which accelerates invasive outgrowth of the cancer through the binding of integrin-β1 on the cell surface. However, whether this machinery is subject to the LOXL4-mediated intrusive regulation remains uncertain.

**Methods:**

Cell invasion was assessed using a transwell-based assay, protein–protein interactions by an immunoprecipitation–Western blotting technique and immunocytochemistry, and plasmin activity in the cell membrane by gelatin zymography.

**Results:**

We revealed that cell surface annexin A2 acts as a receptor of plasminogen via interaction with S100A10, a key cell surface annexin A2-binding factor, and S100A11. We found that the cell surface annexin A2/S100A11 complex leads to mature active plasmin from bound plasminogen, which actively stimulates gelatin digestion, followed by increased invasion.

**Conclusion:**

We have refined our understanding of the role of LOXL4 in TNBC cell invasion: namely, LOXL4 mediates the upregulation of annexin A2 at the cell surface, the upregulated annexin 2 binds S100A11 and S100A10, and the resulting annexin A2/S100A11 complex acts as a receptor of plasminogen, readily converting it into active-form plasmin and thereby enhancing invasion.

## 1 Introduction

In the last few years, breast cancer has been the most prevalent cancer among all cancer types worldwide (1). Triple-negative breast cancer (TNBC) is particularly formidable because of its advanced metastatic aggressiveness and drug resistance (2). Because treatment for TNBC is more limited than that for other types of breast cancer, a deeper understanding of the nature of this type of cancer at both the cellular and molecular levels is required to establish genuinely effective treatments (3–5).

Our recent efforts have shown that lysyl oxidase-like 4 (LOXL4) may be a potent new target for TNBC treatment (6, 7). LOXL4 is a secretory lysyl oxidase enzyme and one of the five members of the LOX family of proteins (LOX; LOXL1–4); these proteins enable cross-bridging among extracellular matrices such as collagen and are closely involved in the formation of cancer-growing stroma (8–10). In our study, we identified a new role of the LOXL4 secreted in TNBC progression: in addition to its traditional collagen cross-linking function, it also targets cancer cell surface annexin A2 as a substrate for enzymatic cross-linking modification. The LOXL4-mediated cross-bridging involves annexin A2 polymerization on the membrane, with the polymerized protein in turn binding to integrin-β1, eventually leading to cell surface accumulation of integrin-β1 at a significant level (7). The process plays a critical role in cancer cell adhesion to tissues, enabling accelerated proliferation and invasion.

However, considering that annexin A2 has many binding partners other than integrin-β1 (11), other proteins binding with annexin A2 on the cell surface may cooperate in these cancer-proliferative events (12–16). There are reports that cell surface annexin A2 acts as a potent plasmin receptor by fashioning a complex with S100A10, an EF-hand type Ca^2+^-binding small protein (around 10 kDa) belonging to the S100 family of proteins, whose cell surface machinery plays a pivotal part in cancer cells to promote metastasis (17–19). This machinery is anticipated to work cooperatively with integrin-β1; however, S100A10 is not drastically elevated compared to normal tissues in invasive breast cancer tissue specimens. Another well-known interaction of annexin A2 with the S100 family of proteins is the interaction with S100A11 (20, 21). Jaiswal et al. (22) determined that annexin A2–S100A11 interaction within cells facilitates plasma membrane repair at the trauma sites that actively enhance cancer invasiveness in TNBC cells. This interaction may arise on the cell surface in part, eliciting the recruitment of plasmins and switching the complex to invasive mode. In this study, we aimed to investigate these postulated mechanisms in detail.

## 2 Materials and methods

### 2.1 Cells and chemicals

The following cells were used in this study: MDA-MB-231 (a human TNBC cell line; ATCC, Rockville, MD, USA), MDA-MB-436 (a human TNBC cell line, ATCC), BT-549 (a human TNBC cell line, ATCC), and HCC3153 [a human TNBC cell line; kindly provided by Dr. Adi F. Gazdar (Hamon Center for Therapeutic Oncology Research and Department of Pathology, the University of Texas Southwestern Medical Center at Dallas, Dallas, TX, USA]. MCF-7 (a human luminal non-TNBC cell line; ATCC) and HEK293T cells (a human embryonic kidney cell line stably expressing the SV40 large T antigen; RIKEN BioResource Center, Tsukuba, Japan) were also used. The stable transformant overexpressing the LOXL4 wild type originating from MDA-MB-231 cells (named MDA-MB-231 LOXL4 wt) has been reported previously (6). The MDA-MB-231 LOXL4 knock-out (KO) subline was also the same as that previously reported (7). All cell lines were cultivated in DMEM/F12 medium (Thermo Fisher Scientific, Waltham, MA, USA) supplemented with 10% fetal bovine serum (FBS). Tranexamic acid (TXA) and MMP9 inhibitor [MMP-9-IN-1 (OUN87710)] were purchased from LKT Laboratories (St. Paul, MN, USA) and Selleck Chemicals (Houston, TX, USA), respectively.

### 2.2 Plasmid constructs

The mammalian gene expression constructs used in this study were all made using the pIDT-SMART-C-TSC vector (pCMViR-TSC) as the backbone to express the cargo genes at significantly high levels (23). The cDNAs located on the multi-cloning site of the pCMViR-TSC were designed to be expressed in a C-terminal 3Myc-6His-tagged or 3HA-6His-tagged form. cDNAs encoding S100A11 or annexin A2 (ANXA2) were inserted into the multi-cloning site of the pCMViR-TSC. Transient transfection of these plasmids into cultured cells was performed using FuGENE-HD (Promega BioSciences, San Luis Obispo, CA, USA).

### 2.3 RNA interference

Stealth RNAi siRNA targeting human S100A11 (siS100A11, ID: HSS109441) and Med GC, a Stealth RNAi siRNA negative control (siCont), were purchased from Thermo Fisher Scientific. Transfection of siRNA (final concentration of 12.5 nM) was performed using Lipofectamine RNAiMAX transfection reagent (Thermo Fisher Scientific).

### 2.4 Purified proteins

The purified proteins, plasminogen (PLG), and tissue-type plasminogen activator (tPA) from human plasma were purchased from Merck Sigma-Aldrich (St. Louis, MO, USA). Biotinylated (bio)-PLG was prepared using a biotinylation kit (Dojindo, Kumamoto, Japan). The glutathione *S*-transferase (GST) fusion proteins were prepared as previously reported (24, 25). In brief, the human annexin A2 (ANXA2), S100A10, and S100A11 cDNAs were cloned into pGEX6P1 vectors (GE Healthcare, Chicago, IL, USA). The cloned pGEX6P1 vectors were transduced into *Escherichia coli*, and the expressed products were purified using glutathione beads (Glutathione Sepharose™ 4 Fast Flow, GE Healthcare). The GST-S100A10 and GST-S100A11 fusion proteins were cleaved by PreScission*™* Protease (GE Healthcare), and the cleaved GST was removed using glutathione beads.

### 2.5 Immunoprecipitation, pull-down, Western blotting, and dot blot

An immunoprecipitation (IP) assay of the expressed foreign proteins was performed using anti-HA tag antibody-conjugated agarose beads (#A2095, Merck Sigma-Aldrich, St. Louis, MO, USA). To examine an active-form transition of plasminogen on the cell surface of TNBC cells, a pull-down of bio-PLG from its treated cell extracts lysed by M-PER cell lysis buffer (Thermo Fisher Scientific) was also investigated using streptavidin-agarose beads (#20219, Thermo Fisher Scientific). Half of the cell lysates were input to confirm the aimed proteins. The remaining half was subjected to IP and pull-down procedures using the above beads. Western blotting (WB) analysis was performed under conventional conditions. In brief, cell lysates were prepared using M-PER cell lysis buffer (Thermo Fisher Scientific). They were supplemented with sodium dodecyl sulfate (SDS)–sample buffer, and 10 µg of the samples was subjected to electrophoresis on SDS–polyacrylamide gel electrophoresis (SDS-PAGE) gel. The proteins separated according to their molecular masses were then transferred onto a polyvinylidene difluoride (PVDF) membrane (Thermo Fisher Scientific) using a semi-dry blotter (Nihoneido, Tokyo, Japan). The membrane was incubated with a blocking buffer [10% skim milk, 6% glycine, and 0.1% Tween-20 in phosphate-buffered saline (PBS)] and then exposed to primary antibodies. The WB was repeated three times for each set of samples. The antibodies used were as follows: mouse anti-HA tag antibody (1:1,000 dilution, #2367, clone 6E2; Cell Signaling Technology, Danvers, MA, USA), mouse anti-Myc tag antibody (1:1,000 dilution, #2276S, clone 9B11; Cell Signaling Technology), mouse anti-human LOXL4 monoclonal antibody (1:1,000 dilution, #sc-374121, Santa Cruz Biotech, Santa Cruz, CA, USA), rabbit anti-human annexin A2 monoclonal antibody (1:1,000 dilution, #8235S, Cell Signaling Technology), and rabbit anti-human MMP9 antibody (1:1,000 dilution, #47449S, Proteintech, Rosemont, IL, USA). For the dot blot assay, the purified proteins (10 µg) were aspiratory blotted onto the nitrocellulose membrane (Thermo Fisher Scientific) in a duplicate manner. The membrane was incubated with a blocking buffer [5% bovine serum albumin (BSA), 6% glycine, 1 mM Ca^2+^, and 0.1% Tween-20 in 50 mM Tris-HCl buffer (pH 7.4)] and then exposed to bio-PLG (10 µg) under the presence or absence of S100A10 or S100A11 (10 µg). The bindings of bio-PLG to the blotted proteins were evaluated using the horseradish peroxidase (HRP)-conjugated streptavidin reagent.

### 2.6 Invasion assay

Cell invasion was evaluated using a Boyden chamber assay with a Matrigel-coated transwell membrane filter insert (pore size, 8 μm) in a 24-well plate (BD Biosciences, Franklin Lakes, NJ, USA). Before the assay, cells were starved in low-serum (0.5% FBS) DMEM/F12 medium. Cells (2 × 10^4^ cells/insert) were seeded with low-serum (0.5% FBS) DMEM/F12 medium in the upper chamber, while the lower chamber was filled with DMEM/F12 medium containing 10% FBS. After incubation for 12 h, cells that passed through the filter were counted by staining with a hematoxylin and eosin (H&E) solution. Cell invasion was imaged under a microscope (BZ-9000; Keyence, Tokyo, Japan) and quantified by cell counting in five non-overlapping fields at ×100 magnification. The numbers of the membrane-passing cells are presented as the average of three independent experimental results.

### 2.7 Chromogenic plasmin assay and gelatin zymography

Membrane protein enrichment was performed using a membrane fractionation kit (Mem-PER™ Plus Membrane Protein Extraction Kit; Thermo Fisher Scientific). The prepared membrane fraction extracts were applied to a chromogenic plasmin activation assay (26, 27) or gelatin zymography. According to the manufacturer’s directions, the plasmin chromogenic evaluation was performed using the measurement assay kit SPECTROZYME^®^ PL (BioMedica Diagnostics Inc., Windsor, NS, Canada). In brief, the assay reaction was performed in the formula of membrane extract 0.01 µg/µL and SPECTROZYME^®^ PL solution 1 mM (total volume 100 µL), which was then incubated at 37°C for 3 h. The plasmin activity was finally determined by measuring the samples’ absorbance increase at a wavelength of 405 nm. In the gelatin zymography, the membrane protein extracts were mixed with non-reducing SDS–sample buffer with no dithiothreitol (DTT) and applied to SDS-PAGE using 8% polyacrylamide gel with 0.5% gelatin. After electrophoresis, SDS was removed via incubation in 2% Triton X-100 at 37°C for 30 min. The gel was then transferred to 0.05 M Tris-HCl buffer (pH 8.0), incubated at 37°C for 18 h, and stained with 1% Coomassie brilliant blue R-250 (FUJIFILM Wako Pure Chemical, Osaka, Japan). The gelatinolytic activity was detected as clear bands against a blue background of undegraded substrate. The activity of matrix metalloproteases (MMPs) was assessed in a similar manner, except that the sample preparation was a 10-fold condensed cell conditioned medium, and the gel reaction buffer was 0.05 M Tris-HCl buffer (pH 8.0) containing 5 mM CaCl_2_.

### 2.8 Immunofluorescence

The live cells grown on the cover glasses were treated with bio-PLG or rabbit anti-human S100A11 polyclonal antibody (1:100 dilution, #10237-1-AP, Proteintech) without fixation for 60 min. After the treated live cells were washed with the conditioned medium, they were fixed with 4% paraformaldehyde (PFA) and stained with the fluorescence-labeled streptavidin (1:200 dilution, #S32356, Thermo Fisher Scientific) or anti-rabbit IgG secondary antibody (goat anti-rabbit IgG [H+L] and the highly cross-adsorbed secondary antibody, Alexa Fluor™ 594 or Alexa Fluor™ 488; 1:200 dilution, #A11012 or #A21441, Thermo Fisher Scientific).

### 2.9 Animal experiment

The animal experimental protocols were approved by the Animal Experiment Committee of Okayama University (approval no. OKU-2020001). All of the mouse procedures and euthanasia, including cell transplantations, were performed painlessly or with the mouse under anesthesia according to the strict guidelines of the University’s Experimental Animal Committee. MDA-MB-231 GFP, a control clone that expresses GFP stably (8 × 10^5^ cells in 0.1 mL PBS/mouse) with TXA (0.5 g/kg) or without them (control PBS), was injected into dorsal tail veins of 7-week-old BALB/c-nu/nu immunocompromised mice. Four mice were injected per group. Thirty-one days later, the rate of metastasized cancer cells in the whole lung was determined by counting the cancer-based white foci (more than 1 mm in diameter).

### 2.10 Statistical analysis

Each experiment was repeated three times, and the resulting raw data were statistically analyzed. The calculated values are means ± standard deviations (SDs). A simple pairwise comparison with Student’s *t*-test was used. In addition, a one-way analysis of variance (ANOVA) was performed for the comparative evaluation of more than two groups. When the ANOVA shows a significant difference, the Bonferroni procedure was used as a *post hoc* test. Probability (p)-values <0.05 were considered statistically significant.

## 3 Results

### 3.1 S100A11 allows the interaction of annexin A2 with plasminogen

**Figure 1:**
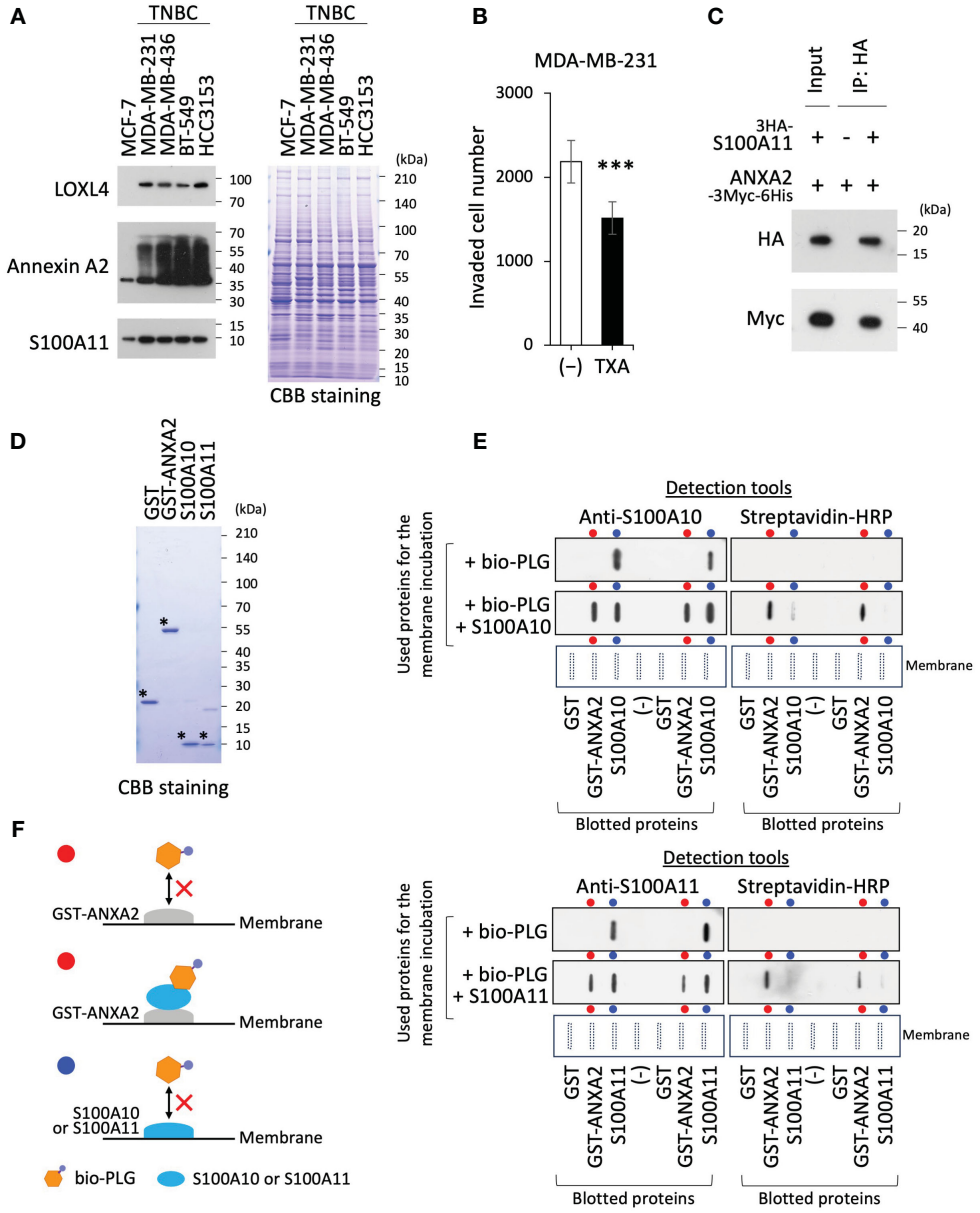
Annexin A2 can bind with plasminogen by fashioning a complex with S100A11. **(A)** Endogenous lysyl oxidase-like 4 (LOXL4), annexin A2, and S100A11 proteins in the indicated cell lines were detected using the Western blotting (WB) procedure. The running gel was also stained with Coomassie brilliant blue (CBB) as a sample control of proper loading. **(B)** We evaluated the invasion ability of MDA-MB-231 cells after the treatment with or without tranexamic acid (TXA) (1 mg/mL). **(C)** HEK293T cells were co-transfected with HA-tagged S100A11 and Myc-tagged ANXA2. After precipitation of the expressed products with the beads, bound foreign proteins were analyzed by WB using the HA or Myc antibody. **(D)** Prepared recombinant proteins (GST, GST-ANXA2, S100A10, and S100A11) from the *Escherichia coli* expression system were checked for their purity. **(E)** Paired blots of the indicated proteins [GST, GST-annexin A2, and S100A10 (upper) or S100A11 (lower)] were dot-blotted onto the nitrocellulose filter membranes in a duplicate manner. The blotted membranes were then incubated with either biotinylated plasminogen (bio-PLG) alone or a blend of bio-PLG and S100A10 (upper) or S100A11 (lower). After incubation was completed, the bound proteins were detected by anti-S100A10 or anti-S100A11 (left) or streptavidin–horseradish peroxidase (HRP) (right). **(F)** The schematic diagram resumed the results from panel **(E)** The red or blue circles correspond to those highlighted in the results in panel **(E)** Data from panel **(B)** are means ± SD, ***p < 0.001. The individual symbols (*)mean exploit of preparations of the purified recombinant proteins as their predicted sizes.

We first confirmed the upregulation of LOXL4 and multimerization of annexin A2 in TNBC cells, which enabled the abundant presentation of annexin A2 on the cell surface. In addition, the expression of S100A11 was slightly elevated in TNBC cells compared to that in non-invasive and non-TNBC MCF-7 cells (**Figure 1A**). Since the invasive activity of MDA-MB-231 cells is significantly dampened by the clinically used plasmin inhibitor TXA (**Figure 1B**), we considered that the LOXL4-mediated cell surface annexin A2 acts as plasmin anchorage through binding with S100A10, which has been reported elsewhere. However, the S100A10 expression level was not highly noticeable in the invasive breast cancers in our *in silico* analysis; rather S100A11, S100A14, and S100P were markedly elevated in the cancer tissues compared to those in normal breast tissues (**Supplementary Figure 1A**). Among these elevated proteins, S100A11 alone was significantly correlated with a cancer-relevant lower survival when it was expressed at consistently high levels under various conditions (**Supplementary Figures 1B, C**). Moreover, it has been well established that annexin A2 interacts with both S100A11 and S100A10 in cells, which we also confirmed (**Figure 1C**). Taking these results together, we presumed that within invasive breast cancer environments, cell surface annexin A2 on the TNBC cells prefers interaction with the highly expressed S100A11 to that with S100A10; this complex then functions as a receptor of plasminogen, where the immature type is transformed into active plasmin. Plasmin is a functional extracellular protease that digests fibrin and collagen, enhancing cancer invasiveness. To confirm this, we prepared four purified recombinant proteins (control GST, GST-annexin A2, S100A10, and S100A11) (**Figure 1D**) and applied them to an interaction study employing protein-blotted membranes. The blotted membrane with a series of recombinant protein duplicates, as indicated at the horizontal bottom layer, was incubated with biotinylated-plasminogen (bio-PLG) or with a combination of bio-PLG and S100A10 (**Figure 1E**, top panel) or S100A11 (**Figure 1E**, bottom panel). Notably, S100A11 displayed the same trend as S100A10: that is, although we detected no bio-PLG interaction with GST-annexin A2 in the presence of bio-PLG alone, bio-PLG was able to bind GST-annexin A2 when S100A10 or S100A11 was also present (**Figure 1E**). We also confirmed the presence of the incubated S100A10 or S100A11 on the annexin A2-blotted lane. The interactive relation among the proteins used was schematically diagramed in **Figure 1F** (the red and blue circles correspond to those highlighted in the results of **Figure 1E**).

### 3.2 Plasminogen is attached to the cell surface of TNBC cells, which matches the cell surface S100A11 location

**Figure 2:**
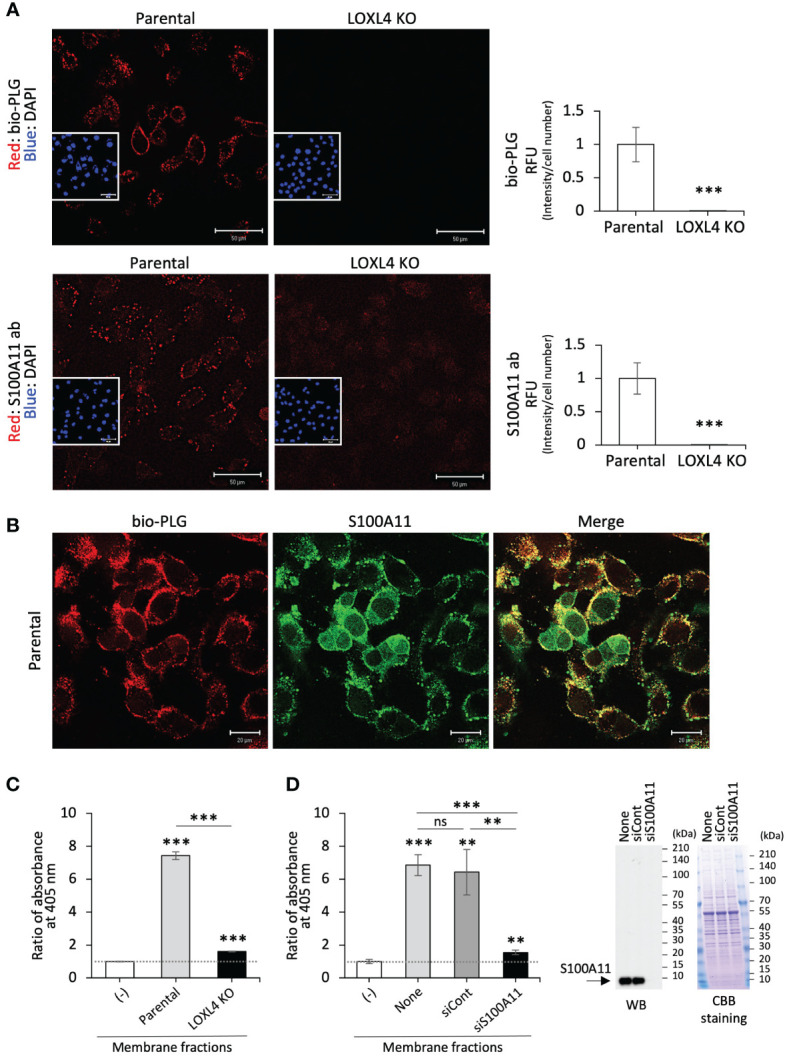
Co-localization of the cell surface S100A11 with plasminogen. **(A)** The indicated live cells were treated with biotinylated plasminogen (bio-PLG) or anti-S100A11 antibody to detect the bio-PLG attached to the cell surface or S100A11 intrinsically residing on the cell surface. Representative images **(A)** are shown, and their signals are quantified. RFUs, relative fluorescent units. **(B)** The live parental MDA-MB-231 cells were treated with both bio-PLG and anti-S100A11 antibodies, and the bio-PLG and anti-S100A11 antibodies bound to the cell surface together were detected simultaneously by different fluorescence colors. **(C)** The membrane fractions prepared from the indicated cells were measured for plasmin activities following a chromogenic plasmin assay method. The absence of the membrane fraction (−) was used as a control for calibration of the assessment. **(D)** Likewise, plasmin activities in the membrane fractions from the parental MDA-MB-231 cells treated with the indicated siRNAs were measured (left). None: MDA-MB-231 cells not treated with any siRNA. The reduced expression of the intrinsic S100A11 was confirmed by Western blotting (WB) (right). Data from panels **(A, C, D)** are means ± SD. ns: not significant, **p < 0.01, ***p < 0.001.

To examine the fashioning of annexin A2, S100A11, and plasminogen into their protein complex on the cell surface of TNBC cells, bio-PLG (**Figure 2A**, top) or anti-S100A11 antibody (S100A11 ab) (**Figure 2A**, bottom) was added to MDA-MB-231 or its intrinsic LOXL4 gene-ablated KO cell culture. The treated living cells were then fixed, and cell-attached bio-PLG or S100A11 ab was visualized with fluorescence-labeled streptavidin (**Figure 2A**). The materials used, i.e., bio-PLG and S100A11, were both detected on the cell membrane as dots, and the signals in both cases were commonly reduced to almost non-detectable levels. Bio-PLG added to the MDA-MB-231 cell culture was co-localized with S100A11 in most parts of the cell membrane before fixation treatment of the cells, which was also confirmed (**Figure 2B**). It was confirmed that the PLG attached to the plasma membrane surface leads to plasmin activity, which is much higher in parental cells than in the LOXL4 KO cells (**Figure 2C**) (7). In addition, the manifestation of enzymatic active-form plasmin from the PLG in the membrane fraction required S100A11 since siRNA-mediated lowered expression of S100A11 markedly obstructed the plasmin activity (**Figure 2D**). To expand on these findings, a clinically prepared invasive breast cancer specimen was stained for plasminogen. As a result, a positive signal of plasminogen, apparently with the membrane localization, was observed in the LOXL4/annexin A2/S100A11-positive case (**Supplementary Figure 2A**).

### 3.3 Plasminogen turns into active-form plasmin on the attached ground of the cell surface annexin A2/S100A11 complex in TNBC cells

**Figure 3:**
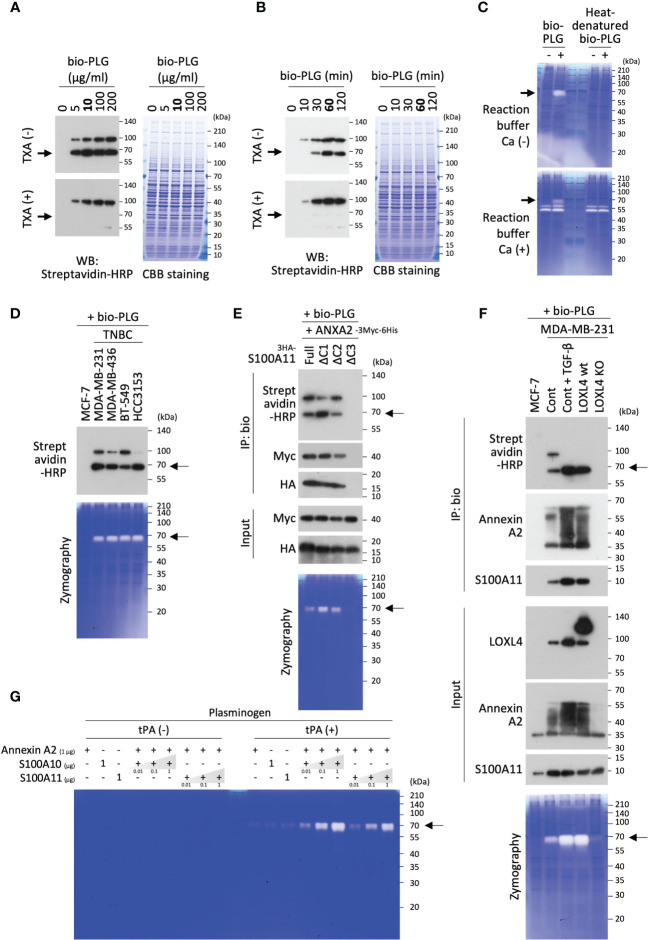
Transition of plasminogen to active-form plasmin on the cell surface annexin A2/S100A11 complex. **(A)** Biotinylated plasminogen (bio-PLG) was evaluated for the dose used in its transition reaction to active-form plasmin in MDA-MB-231 cell culture. A treatment duration of 60 min was scheduled. To confirm the ripe plasmin band originating from the added bio-PLG, its reaction stopper tranexamic acid (TXA) (1 mg/mL) was used. **(B)** The temporal evaluation of the added bio-PLG was performed according to a protocol similar to that described in the former experiment **(A)**, except for using only 10 µg/mL. **(C)** The effect of calcium (Ca^2+^, 10 mM) on plasmin and other protease activities was studied. The samples prepared from the cell membrane fractions under non-reducing [dithiothreitol (DTT) (−)] conditions were subjected to gelatin zymography. After running, the gels were soaked in a refolding buffer and then incubated with a reaction buffer with (lower) or without Ca^2+^ (upper). **(D)** Non-triple-negative breast cancer (TNBC) MCF-7 cells and the indicated TNBC cell lines were all treated with bio-PLG at a final concentration of 10 µg/mL for 60 min. The bio-PLG attached to the cell membrane and the manifestation of its active derivative in the individual cell membranes were detected by Western blotting (WB) and zymography. **(E)** HEK293T cells were co-transfected with HA-tagged full-length or any of several S100A11 variants lacking the C-terminal end (see **Supplementary Figure 2C**) and Myc-tagged annexin A2. The transfected cells were further treated with bio-PLG (10 µg/mL, 60 min). After fractionation of the membrane compartment from each cell treated, half of the extracts were used for input detection and zymography experiments. The remaining half was subjected to a pull-down procedure using streptavidin-conjugated beads. After precipitation of the samples with the beads, bound proteins were analyzed by WB using streptavidin–horseradish peroxidase (HRP), HA antibody, or Myc antibody. **(F)** The indicated cells were all treated with bio-PLG (10 µg/mL, 60 min). TGF-β (10 ng/mL, 24 h) stimulation induced intrinsic lysyl oxidase-like 4 (LOXL4) production in the parental MDA-MB-231 cells. After fractionation of the membrane compartment from each cell treated, similar experiments were performed as in panel **(E)** except for the use of the indicated antibodies to evaluate the endogenous proteins of interest. Cont: non-engineered MDA-MB-231 cells. **(G)** *In vitro*, a cell-free reaction was performed to evaluate the effect of annexin A2/S100A11 complex on the emergence of plasmin from inactive plasminogen. The prepared, purified proteins were mixed and incubated according to the formula. After a 30-min reaction at room temperature, the samples were subjected to gelatin zymography.

To catalyze protease activity, plasminogen must transit into active-form plasmin. We examined whether the operational transformation of plasminogen occurred on the cell surface annexin A2/S100A11 complex as on the annexin A2/S100A10 complex reported elsewhere (12, 17, 18, 20, 28). It was found that the transition reaction did arise in MDA-MB-231 cells when bio-PLG was poured onto the cell culture (**Figure 3A**). Evaluation of the dose and timing of the addition of bio-PLG to MDA-MB-231 cell culture showed that the transition to active form was saturated at 10 µg/mL (**Figure 3A**), and the reaction culminated at 60 min for the 10 µg/mL dose (**Figure 3B**). In these figures, the active-form transition was confirmed by the manifestation of a cleaved band size slightly below 70 kDa compared to immature plasminogen (with a band at nearly 100 kDa), matching the known molecular weight of mature plasmin. In addition, it was confirmed that the cleaved product was the active-form plasmin in the gelatin zymography images using the MDA-MB-231 membrane fractions that were treated or not treated with bio-PLG (**Figure 3C**). Owing to the precise digested-band detection by the plasmin formed in the membrane fraction prepared under the run-gel incubation with calcium absence reaction buffer (**Figure 3C**, top), the subsequent zymography experiments were performed as calcium-adverse gel reactions except when detection of MMP activity required calcium in the reaction buffer.

According to the experimental conditions above, bio-PLG was added to several breast cancer cell lines, and their plasmin activities were evaluated. This was followed by the detection of the membrane-attached full-length PLG to the TNBC cells and constant digestive transition to the active-form plasmin (**Figure 3D**). The event was not observed in the non-invasive and non-TNBC cell line MCF-7 cells, which had no LOXL4 and no multimerized annexin A2 as shown in **Figure 1A**. It was shown that the interaction of plasminogen and its cell surface receptors, including S100A10 and a few cationic amino acids with several different patterns, plays a crucial role in plasminogen capture. This led us to query whether the C-terminal cationic amino acids of S100A11 are significant for the interaction between plasminogen and annexin A2. Several C-terminal-deletion constructs, as indicated in **Supplementary Figure 2C**, were evaluated for plasminogen attachment and its dynamics relative to the active form. The results were similar to those for the previously reported receptors: C-terminal cationic amino acids, particularly the serial alignment of lysin and arginine, were found to be required for the interaction with plasminogen (**Figure 3E**). To further elucidate the relationship among LOXL4, annexin A2, S100A11, and plasminogen, an immunoprecipitation-based interaction assay among them was performed, together with zymography using various cell types: LOXL4-negative MCF-7 cells, non-treated intact MDA-MB-231 cells, TGF-β-treated MDA-MB-231 cells (in which intrinsic LOXL4 is highly induced), ectopic LOXL4 wt-overexpressed MDA-MB-231 cells, and inherent LOXL4 gene-ablated MDA-MB-231 cells. As shown in **Figure 3F**, it was found that bio-PLG was co-immunoprecipitated with both multimerized annexin A2 and S100A11 in the intact MDA-MB-231 cells (lane 2) but not in the MCF-7 cells (lane 1). The interaction was further enhanced in both TGF-β-treated MDA-MB-231 (lane 3) and LOXL4 wt-overexpressed MDA-MB-231 cells (lane 4), in which annexin A2 multimerization and the transition of plasminogen to active-form plasmin were markedly elevated compared to those in non-treated and non-gene-engineered parental cells (lane 2). Those events did not occur in the LOXL4-KO cells (lane 5) as in MCF-7 cells (lane 1). The S100A11-relevant plasminogen transition to active-form plasmin was finally confirmed by an *in vitro* cell-free experiment (**Figure 3G**). Like plasminogen, the cell surface S100A10 could trap tPA, which facilitates plasmin generation by accessing its neighboring plasminogen; hence, tPA was added or not added to the reaction mixture of proteins composed of plasminogen, annexin A2, and S100A11 or S100A10 as a positive control, and incubation was performed accordingly. By this approach, it was revealed that S100A11, as well as S100A10, contributes to the production of plasmin from immature plasminogen. Cumulatively, these results indicate that, like S100A10, S100A11 plays a significant role as a plasminogen receptor by binding with cell surface annexin A2, which in turn efficiently generates plasmin on the TNBC cell surface.

### 3.4 Cell surface plasmin promotes upregulation of invasion of TNBC cells, which links to lung metastasis

**Figure 4:**
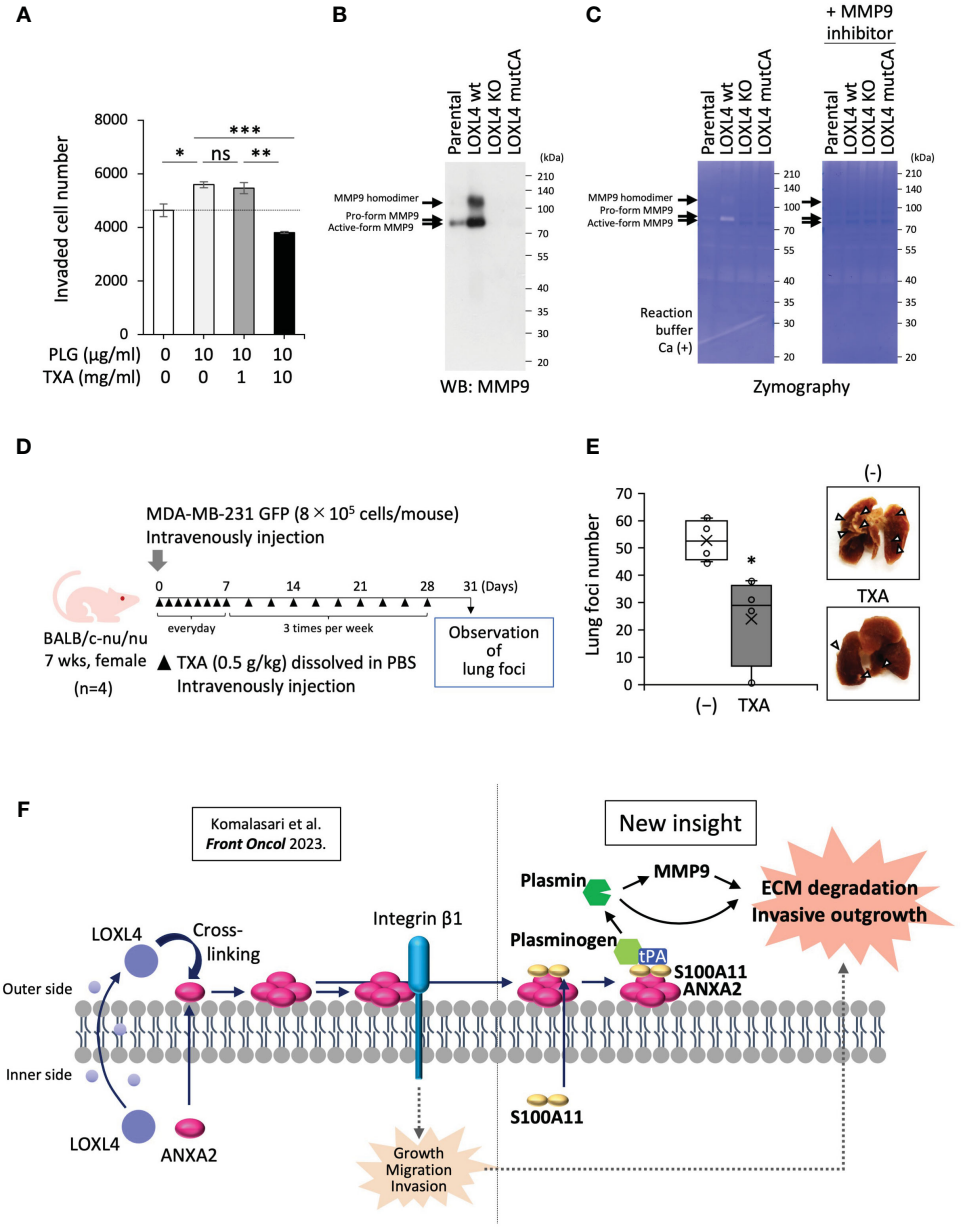
Contribution of cell surface plasmin to activation of MMP9 and invasion of cancer cells. **(A)** A transwell-based invasion assay was performed on MDA-MB-231 cells treated with plasminogen (PLG; 10 µg/mL, 12 h) under the presence or absence of tranexamic acid (TXA) (1 or 10 mg/mL). **(B)** Prepared conditioned media from 1-day serum-free cultivation of the indicated cells were condensed 10-fold. The media specimens were subjected to sodium dodecyl sulfate–polyacrylamide gel electrophoresis (SDS-PAGE) and then underwent Western blotting (WB) analysis to detect MMP9 after being transferred to a polyvinylidene difluoride (PVDF) membrane or Coomassie brilliant blue (CBB) staining of the running gel without the transfer procedure, which was used as a sample control for proper loading. **(C)** Zymography using the gelatin substrate was performed to detect MMP-secretory activity from the indicated cells. **(D)** Schematic representation of the *in vivo* experimental lung tropic cancer metastasis protocol. MDA-MB-231 GFP cells (8 × 10^5^ cells) with or without TXA (0.5 g/kg) were injected intravenously into BALB/c-nu/nu female mice. **(E)** Lung metastasis was monitored by images (right) from cancer-based white foci in the dissected mouse lung, and the clear foci (left) were counted as total foci (more than 1 mm in diameter). **(F)** Schematic representation of the molecular interplay among the indicated vital molecules. Data from panels **(A, E)** are means ± SD. ns: not significant, *p < 0.05, **p < 0.01, ***p < 0.001.

Next, we asked whether the plasmin production mediated by annexin A2/S100A11 complex on the cell surface of the LOXL4-positive TNBC cells promotes cancer metastasis. The transwell chamber-based invasion assay showed that supplementation of MDA-MB-231 cell culture with plasminogen enhanced invasion activity, and this enhancement was significantly dampened by the presence of TXA (**Figure 4A**). The cancer-invasive activity is regulated by several MMPs, among which MMP9 can be processed by plasmin, resulting in the manifestation of active MMP9. To further investigate the mechanism underlying the enhanced invasive activity, we prepared condensed cell conditioned media from MDA-MB-231 cells and their gene-engineered clones [ectopic LOXL4 wt or LOXL4 mutCA (catalytically inactive mutation)-overexpressed MDA-MB-231 cells and inherent LOXL4 gene KO MDA-MB-231 cells]. Interestingly, we found that MMP9 induction at both the protein level (**Figure 4B**) and the enzymatic activity (**Figure 4C**) was much higher from the LOXL4 wt-overexpressed cells than from the parental cells. These events were diminished in the media prepared from LOXL4 KO cells or LOXL4 mutCA-overexpressed cells, suggesting that cell surface plasmin cooperatively works with MMP9 to achieve digestion of the extracellular matrix of TNBC cells. We finally evaluated the metastatic role of the cell surface plasmin using an *in vivo* metastasis mouse model (**Figure 4D**) by which we found that the lung-tropic metastasis was significantly stalled by the treatment with TXA (**Figure 4E**), suggesting an active involvement of plasmin in the breast cancer metastasis. Metastasis regulated by our identified plasmin-activating machinery may be life-threatening. It is known that high protein levels of S100A10, S100A11, annexin A2, and/or plasminogen are associated with low survival in breast cancer patients (**Supplementary Figure 2D** **).** Finally, we schematized the new machinery as the unique insight shown in **Figure 4F**: multimerized annexin A2 acts as a plasminogen receptor with the help of S100A11 as well as S100A10, leading to plasmin generation. The abundant cell surface plasmin on TNBC cells cooperatively works with activated MMP9 for extracellular matrix (ECM) digestion, eventually promoting invasive metastasis.

## 4 Discussion

Adding to the previous finding about the new role of LOXL4 in TNBC cell progression, this study revealed that the LOXL4-mediated multimerized annexin A2 on the cell surface functions as a receptor of plasminogen with the help of S100A11 as well as the classical factor S100A10, which facilitates the transition of rudimentary plasminogen to active-form plasmin. The cell surface abundant plasmin formed by the identified pathway ultimately contributes to invasiveness in the TNBC cells. The multimerized annexin A2 also coordinates with abundant integrin-β1 on the cell surface, allowing the growth, migration, and invasion of TNBC cells. The newly identified machinery of plasmin activation works cooperatively with the integrin-β1-mediated pathway we formerly revealed (7), particularly for the invasion event.

The interaction of annexin A2 and S100A11 within TNBC cells has already been reported: the complex functions in the plasma membrane repair that facilitates cell invasion (22). However, to our knowledge, the cell surface interaction of annexin A2 and S100A11 has never been reported prior to this study. Thus, TNBC cells can accelerate their invasion with multiple mechanisms, even with the single molecule annexin A2 (29–31). It has been reported that the cell surface presentation of annexin A2 requires Src-mediated tyrosine phosphorylation, which is readily elevated in TNBC cells (32–34). The extracellularly secreted LOXL4 catalytically works to lock annexin A2 on the cell membrane surface by multimerization modification. Importantly, the intracellular S100A11 moving to the cell surface has already formed a complex with annexin A2. Otherwise, the S100A11 secreted from TNBC would bind to the cell surface annexin A2 in an autocrine manner. We previously demonstrated that S100A11 is actively secreted from several types of cancer cells (35), and the secreted S100A11 acts to fuel cancer progression (36, 37). Owing to the elevated expression of S100A11 compared to S100A10 in TNBC, chances are that cell surface annexin A2 binds with S100A11 as well as S100A10.

Another point worth discussing is that the function of plasmin is not restricted to ECM digestion. Plasmin does play a role in activating MMP9 (**Figures 4B, C**), suggesting the presence of the orchestrated machinery of ECM digestion by the cell surface plasmin with MMP9 that enhances the invasive activity of TNBC cells (38). In addition, plasmin contributes to the generation of mature active-type TGF-β from the rudimentary or “latent” TGF-β (39). TGF-β is a key factor inducing cancer cell invasion by the epithelial–mesenchymal transition (EMT) (40). Thus, cell surface plasmin promotes TNBC cell invasion through multiple mechanisms. Our *in silico* analysis explains the manifestation of significantly lower survival in patients with breast cancers with high levels of plasminogen protein (**Supplementary Figure 2D**).

As described above, these findings open up the possibility that a potent plasmin inhibitor, TXA, may work to stall TNBC cell invasion. Our transwell-based cell invasion evaluations showed that TXA significantly dampened invasion of the TNBC cell placed in the upper chamber with the low-concentration (0.5%) FBS medium (**Figure 1B**) even when excess plasminogen from a foreign source was present in the upper chamber medium at much higher than the native dose (i.e., 0.5% FBS supplemented with DMEM/F-12 medium) (**Figure 4A**). A higher concentration of TXA was required to inhibit invasion in the latter case. It has been reported that the milk protein lactoferrin works to prevent cancer progression through iron chelation by competing with transferrin, which is in the same family of proteins (41, 42). Owing to the solid inhibitory power of lactoferrin against plasmin (43, 44), its cancer prevention effect may be ascribed to both chelation of iron (a vital cancer nutrient) and inhibition of plasmin. These results emphasize that cell surface plasmin is a potent druggable target for the prevention of life-threatening cancer metastasis. However, considering the daily physiological significance of plasmin in our body, we cannot say that a plasmin inhibition strategy aiming to suppress its catalytic activity is warranted for use over an extended period. Our identified mechanism provided the options of cell surface annexin A2 and S100A11 as therapeutic target molecules. Still, unfortunately, the abundant cell surface receptors for plasminogen (**Supplementary Figure 2B**) (28) may operate to trap plasminogen as a surrogate molecule for the inhibited receptor molecule. Considering these factors, an inhibitory medicine targeting the plasminogen molecule but not the catalytic activity by which plasminogen is released from cell surface receptors may be optimal. Otherwise, extracellular LOXL4 is an attractive target molecule for TNBC therapy. One previous report has shown that anti-LOXL4 antibody effectively prevents head-and-neck cancer progression (45). This antibody may also be helpful in preventing metastatic progression in TNBC.

## Data availability statement

The raw data supporting the conclusions of this article will be made available by the authors, without undue reservation.

## Ethics statement

Ethical approval was not required for the studies on humans in accordance with the local legislation and institutional requirements because only commercially available established cell lines were used.

## Author contributions

TT: Data curation, Formal analysis, Investigation, Methodology, Writing – original draft, Writing – review & editing. NT: Data curation, Formal analysis, Funding acquisition, Investigation, Methodology, Writing – review & editing. RK: Funding acquisition, Investigation, Writing – review & editing. KY: Investigation, Writing – review & editing. HM: Investigation, Writing – review & editing. NK: Investigation, Methodology, Writing – review & editing. YC: Funding acquisition, Investigation, Methodology, Writing – review & editing. FJ: Investigation, Methodology, Writing – review & editing. YG: Investigation, Writing – review & editing. TO: Investigation, Writing – review & editing. IR: Investigation, Writing – review & editing. IS: Investigation, Writing – review & editing. JZ: Investigation, Writing – review & editing. TH: Investigation, Writing – review & editing. YS: Investigation, Methodology, Writing – review & editing. AY: Data curation, Formal analysis, Writing – review & editing. FK: Data curation, Formal analysis, Writing – review & editing. EK: Data curation, Formal analysis, Writing – review & editing. YI: Investigation, Writing – review & editing. JF: Investigation, Writing – review & editing. ST: Data curation, Formal Analysis, Writing – review & editing. YZ: Investigation, Writing – review & editing. MS: Conceptualization, Data curation, Funding acquisition, Investigation, Methodology, Project administration, Supervision, Writing – original draft, Writing – review & editing.

## References

[1] Kashyap D Pal D Sharma R Garg VK Goel N Koundal D. Global increase in breast cancer incidence: risk factors and preventive measures. BioMed Res Int. (2022) 2022:9605439. doi: 10.1155/2022/9605439 35480139 PMC9038417

[2] Riggio AI Varley KE Welm AL. The lingering mysteries of metastatic recurrence in breast cancer. Br J Cancer. (2021) 124:13–26. doi: 10.1038/s41416-020-01161-4 33239679 PMC7782773

[3] Yersal O Barutca S. Biological subtypes of breast cancer: prognostic and therapeutic implications. World J Clin Oncol. (2014) 5:412–24. doi: 10.5306/wjco.v5.i3.412 PMC4127612 25114856

[4] Moamin MR Allen R Woods SL Brown JE Nunns H Juncker-Jensen A. Changes in the immune landscape of tnbc after neoadjuvant chemotherapy: correlation with relapse. Front Immunol. (2023) 14:1291643. doi: 10.3389/fimmu.2023.1291643 38090569 PMC10715438

[5] Carvalho FM. Triple-negative breast cancer: from none to multiple therapeutic targets in two decades. Front Oncol. (2023) 13:1244781. doi: 10.3389/fonc.2023.1244781 38023167 PMC10666917

[6] Hirabayashi D Yamamoto KI Maruyama A Tomonobu N Kinoshita R Chen Y. Loxl1 and loxl4 are novel target genes of the zn(2+)-bound form of zeb1 and play a crucial role in the acceleration of invasive events in triple-negative breast cancer cells. Front Oncol. (2023) 13:1142886. doi: 10.3389/fonc.2023.1142886 36910659 PMC9997211

[7] Komalasari N Tomonobu N Kinoshita R Chen Y Sakaguchi Y Gohara Y. Lysyl oxidase-like 4 exerts an atypical role in breast cancer progression that is dependent on the enzymatic activity that targets the cell-surface annexin A2. Front Oncol. (2023) 13:1142907. doi: 10.3389/fonc.2023.1142907 37091157 PMC10114587

[8] Xu M Zhang T Xia R Wei Y Wei X. Targeting the tumor stroma for cancer therapy. Mol Cancer. (2022) 21:208. doi: 10.1186/s12943-022-01670-1 36324128 PMC9628074

[9] Xiao Q Ge G. Lysyl oxidase, extracellular matrix remodeling and cancer metastasis. Cancer Microenviron. (2012) 5:261–73. doi: 10.1007/s12307-012-0105-z PMC3460045 22528876

[10] Liburkin-Dan T Toledano S Neufeld G. Lysyl oxidase family enzymes and their role in tumor progression. Int J Mol Sci. (2022) 23:6249. doi: 10.3390/ijms23116249 35682926 PMC9181702

[11] Christensen MV Hogdall CK Jochumsen KM Hogdall EVS. Annexin A2 and cancer: A systematic review. Int J Oncol. (2018) 52:5–18. doi: 10.3892/ijo.2017.4197 29115416

[12] Bharadwaj A Bydoun M Holloway R Waisman D. Annexin A2 heterotetramer: structure and function. Int J Mol Sci. (2013) 14:6259–305. doi: 10.3390/ijms14036259 PMC3634455 23519104

[13] Zhang C Zhou T Chen Z Yan M Li B Lv H. Coupling of integrin alpha5 to annexin A2 by flow drives endothelial activation. Circ Res. (2020) 127:1074–90. doi: 10.1161/CIRCRESAHA.120.316857 32673515

[14] Grindheim AK Saraste J Vedeler A. Protein phosphorylation and its role in the regulation of annexin A2 function. Biochim Biophys Acta Gen Subj. (2017) 1861:2515–29. doi: 10.1016/j.bbagen.2017.08.024 28867585

[15] Lopez-Rodriguez JC Martinez-Carmona FJ Rodriguez-Crespo I Lizarbe MA Turnay J. Molecular dissection of the membrane aggregation mechanisms induced by monomeric annexin A2. Biochim Biophys Acta Mol Cell Res. (2018) 1865:863–73. doi: 10.1016/j.bbamcr.2018.03.010 29567212

[16] Shalhout SZ Yang PY Grzelak EM Nutsch K Shao S Zambaldo C. Yap-dependent proliferation by a small molecule targeting annexin A2. Nat Chem Biol. (2021) 17:767–75. doi: 10.1038/s41589-021-00755-0 33723431

[17] Bharadwaj A Kempster E Waisman DM. The annexin A2/S100a10 complex: the mutualistic symbiosis of two distinct proteins. Biomolecules. (2021) 11:1849. doi: 10.3390/biom11121849 34944495 PMC8699243

[18] Huang D Yang Y Sun J Dong X Wang J Liu H. Annexin A2-S100a10 heterotetramer is upregulated by pml/raralpha fusion protein and promotes plasminogen-dependent fibrinolysis and matrix invasion in acute promyelocytic leukemia. Front Med. (2017) 11:410–22. doi: 10.1007/s11684-017-0527-6 28687976

[19] Li Z Yu L Hu B Chen L Jv M Wang L. Advances in cancer treatment: A new therapeutic target, annexin A2. J Cancer. (2021) 12:3587–96. doi: 10.7150/jca.55173 PMC8120175 33995636

[20] Liu Y Myrvang HK Dekker LV. Annexin A2 complexes with S100 proteins: structure, function and pharmacological manipulation. Br J Pharmacol. (2015) 172:1664–76. doi: 10.1111/bph.12978 PMC4376447 25303710

[21] Ashraf APK Gerke V. The resealing factor S100a11 interacts with annexins and extended synaptotagmin-1 in the course of plasma membrane wound repair. Front Cell Dev Biol. (2022) 10:968164. doi: 10.3389/fcell.2022.968164 36200035 PMC9527316

[22] Jaiswal JK Lauritzen SP Scheffer L Sakaguchi M Bunkenborg J Simon SM. S100a11 is required for efficient plasma membrane repair and survival of invasive cancer cells. Nat Commun. (2014) 5:3795. doi: 10.1038/ncomms4795 24806074 PMC4026250

[23] Sakaguchi M Watanabe M Kinoshita R Kaku H Ueki H Futami J. Dramatic increase in expression of a transgene by insertion of promoters downstream of the cargo gene. Mol Biotechnol. (2014) 56:621–30. doi: 10.1007/s12033-014-9738-0 PMC4067539 24526517

[24] Nukui T Ehama R Sakaguchi M Sonegawa H Katagiri C Hibino T. S100a8/A9, a key mediator for positive feedback growth stimulation of normal human keratinocytes. J Cell Biochem. (2008) 104:453–64. doi: 10.1002/jcb.21639 18044712

[25] Tomonobu N Kinoshita R Wake H Inoue Y Ruma IMW Suzawa K. Histidine-rich glycoprotein suppresses the S100a8/A9-mediated organotropic metastasis of melanoma cells. Int J Mol Sci. (2022) 23:10300. doi: 10.3390/ijms231810300 36142212 PMC9499646

[26] Kassam G Choi KS Ghuman J Kang HM Fitzpatrick SL Zackson T. The role of annexin ii tetramer in the activation of plasminogen. J Biol Chem. (1998) 273:4790–9. doi: 10.1074/jbc.273.8.4790 9468544

[27] Filipenko NR Kang HM Waisman DM. Characterization of the ca2+-binding sites of annexin ii tetramer. J Biol Chem. (2000) 275:38877–84. doi: 10.1074/jbc.M004125200 10980196

[28] Madureira PA O'Connell PA Surette AP Miller VA Waisman DM. The biochemistry and regulation of S100a10: A multifunctional plasminogen receptor involved in oncogenesis. J BioMed Biotechnol. (2012) 2012:353687. doi: 10.1155/2012/353687 23118506 PMC3479961

[29] Chaudhary P Thamake SI Shetty P Vishwanatha JK. Inhibition of triple-negative and herceptin-resistant breast cancer cell proliferation and migration by annexin A2 antibodies. Br J Cancer. (2014) 111:2328–41. doi: 10.1038/bjc.2014.542 PMC4264449 25321192

[30] Mahdi AF Nolan J O'Connor RI Lowery AJ Allardyce JM Kiely PA. Collagen-I influences the post-translational regulation, binding partners and role of annexin A2 in breast cancer progression. Front Oncol. (2023) 13:1270436. doi: 10.3389/fonc.2023.1270436 37941562 PMC10628465

[31] Huang Y Jia M Yang X Han H Hou G Bi L. Annexin A2: the diversity of pathological effects in tumorigenesis and immune response. Int J Cancer. (2022) 151:497–509. doi: 10.1002/ijc.34048 35474212

[32] Fan Y Si W Ji W Wang Z Gao Z Tian R. Rack1 mediates tyrosine phosphorylation of anxa2 by src and promotes invasion and metastasis in drug-resistant breast cancer cells. Breast Cancer Res. (2019) 21:66. doi: 10.1186/s13058-019-1147-7 31113450 PMC6530024

[33] Gibbs LD Mansheim K Maji S Nandy R Lewis CM Vishwanatha JK. Clinical significance of annexin A2 expression in breast cancer patients. Cancers (Basel). (2020) 13:2. doi: 10.3390/cancers13010002 33374917 PMC7792619

[34] Long Y Chong T Lyu X Chen L Luo X Faleti OD. Foxd1-dependent rala-anxa2-src complex promotes ctc formation in breast cancer. J Exp Clin Cancer Res. (2022) 41:301. doi: 10.1186/s13046-022-02504-0 36229838 PMC9558416

[35] Saho S Satoh H Kondo E Inoue Y Yamauchi A Murata H. Active secretion of dimerized S100a11 induced by the peroxisome in mesothelioma cells. Cancer Microenviron. (2016) 9:93–105. doi: 10.1007/s12307-016-0185-2 27334300 PMC5264658

[36] Takamatsu H Yamamoto KI Tomonobu N Murata H Inoue Y Yamauchi A. Extracellular S100a11 plays a critical role in spread of the fibroblast population in pancreatic cancers. Oncol Res. (2019) 27:713–27. doi: 10.3727/096504018X15433161908259 PMC7848439 30850029

[37] Mitsui Y Tomonobu N Watanabe M Kinoshita R Sumardika IW Youyi C. Upregulation of mobility in pancreatic cancer cells by secreted S100a11 through activation of surrounding fibroblasts. Oncol Res. (2019) 27:945–56. doi: 10.3727/096504019X15555408784978 PMC7848232 31046874

[38] Davis GE Pintar Allen KA Salazar R Maxwell SA. Matrix metalloproteinase-1 and -9 activation by plasmin regulates a novel endothelial cell-mediated mechanism of collagen gel contraction and capillary tube regression in three-dimensional collagen matrices. J Cell Sci. (2001) 114:917–30. doi: 10.1242/jcs.114.5.917 11181175

[39] Santibanez JF Obradovic H Kukolj T Krstic J. Transforming growth factor-beta, matrix metalloproteinases, and urokinase-type plasminogen activator interaction in the cancer epithelial to mesenchymal transition. Dev Dyn. (2018) 247:382–95. doi: 10.1002/dvdy.24554 28722327

[40] Hao Y Baker D Ten Dijke P. Tgf-beta-mediated epithelial-mesenchymal transition and cancer metastasis. Int J Mol Sci. (2019) 20:2767. doi: 10.3390/ijms20112767 31195692 PMC6600375

[41] Pan S Weng H Hu G Wang S Zhao T Yao X. Lactoferrin may inhibit the development of cancer via its immunostimulatory and immunomodulatory activities (Review). Int J Oncol. (2021) 59:85. doi: 10.3892/ijo.2021.5265 34533200

[42] Cutone A Rosa L Ianiro G Lepanto MS Bonaccorsi di Patti MC Valenti P. Lactoferrin's anti-cancer properties: safety, selectivity, and wide range of action. Biomolecules. (2020) 10:456. doi: 10.3390/biom10030456 32183434 PMC7175311

[43] Zwirzitz A Reiter M Skrabana R Ohradanova-Repic A Majdic O Gutekova M. Lactoferrin is a natural inhibitor of plasminogen activation. J Biol Chem. (2018) 293:8600–13. doi: 10.1074/jbc.RA118.003145 PMC5986228 29669808

[44] Chea C Miyauchi M Inubushi T Okamoto K Haing S Takata T. Molecular mechanisms of inhibitory effects of bovine lactoferrin on invasion of oral squamous cell carcinoma. Pharmaceutics. (2023) 15:562. doi: 10.3390/pharmaceutics15020562 36839884 PMC9958951

[45] Gorogh T Quabius ES Heidebrecht H Nagy A Muffels T Haag J. Lysyl oxidase like-4 monoclonal antibody demonstrates therapeutic effect against head and neck squamous cell carcinoma cells and xenografts. Int J Cancer. (2016) 138:2529–38. doi: 10.1002/ijc.29986 26756583

