# Supplementary material for: Lysyl oxidase-like 4 promotes the invasiveness of triple-negative breast cancer cells by orchestrating the invasive machinery formed by annexin A2 and S100A11 on the cell surface
Source: Front Oncol. 2024 Mar 26;14:1371342. doi: 10.3389/fonc.2024.1371342 (PMC11002074; doi:10.3389/fonc.2024.1371342)
Supplement: Supplementary file 1 [file DataSheet_1.docx]

Supplementary Material

Lysyl oxidase-like 4 promotes the invasiveness of triplenegative breast cancer cells by orchestrating the invasive machinery formed by annexin A2 and S100A11 on the cell surface

Tetta Takahashi^1,2†^, Nahoko Tomonobu^1†^, Rie Kinoshita^1^, Ken-ichi Yamamoto^1^, Hitoshi Murata^1^, Ni Luh Gede Yoni Komalasari^3^, Youyi Chen^4^, Fan Jiang^1^, Yuma Gohara^1^, Toshiki Ochi^1,5^, I Made Winarsa Ruma^3^, I Wayan Sumardika^3^, Jin Zhou^6^, Tomoko Honjo^7^, Yoshihiko Sakaguchi^8^, Akira Yamauchi^9^, Futoshi Kuribayashi^9^, Eisaku Kondo^10^, Yusuke Inoue^11^, Junichiro Futami^7^, Shinichi Toyooka^12^, Yoshito Zamami^2^, Masakiyo Sakaguchi^1*^

^1^ Department of Cell Biology, Okayama University Graduate School of Medicine, Dentistry and Pharmaceutical Sciences, Okayama, Japan.

^2^ Department of Pharmacy, Okayama University Hospital, Okayama, Japan.

^3^ Faculty of Medicine, Udayana University, Denpasar, Bali, Indonesia.

^4^ Department of Breast Surgery, The First Affiliated Hospital, Zhejiang University School of Medicine, Hangzhou 310003, PR. China.

^5^ Department of Neurology, Okayama University Graduate School of Medicine, Dentistry and Pharmaceutical Sciences, Okayama, Japan.

^6^ Medical Oncology Department of Gastrointestinal Tumors, Liaoning Cancer Hospital & Institute, Cancer Hospital of the Dalian University of Technology, Shenyang, Liaoning, China.

^7^ Department of Interdisciplinary Science and Engineering in Health Systems, Okayama University, Okayama, Japan.

^8^ Department of Microbiology, Tokushima Bunri University, Sagamihara, Tokushima, Japan.

^9^ Department of Biochemistry, Kawasaki Medical School, Kurashiki, Okayama, Japan.

^10^ Division of Tumor Pathology, Near InfraRed Photo-Immuno-Therapy Research Institute, Kansai Medical University, Osaka, Japan.

^11^ Faculty of Science and Technology, Division of Molecular Science, Gunma University, Kiryu, Gunma, Japan.

^12^ Department of General Thoracic Surgery and Breast and Endocrinological Surgery, Okayama University Graduate School of Medicine, Dentistry and Pharmaceutical Sciences, Okayama, Japan

^†^These authors contributed equally to this work and share the first authorship

***Corresponding Author**: Masakiyo Sakaguchi, Ph.D.
E-mail: [masa-s@md.okayama-u.ac.jp](mailto:masa-s@md.okayama-u.ac.jp); Phone number: +81-86-235-7395; Fax: +81-86-235-7400; Department of Cell Biology, Okayama University Graduate School of Medicine, Dentistry and Pharmaceutical Sciences, 2-5-1 Shikata-cho, Kita-ku, Okayama-shi, Okayama 700-8558, Japan

# Supplementary Figures

## *
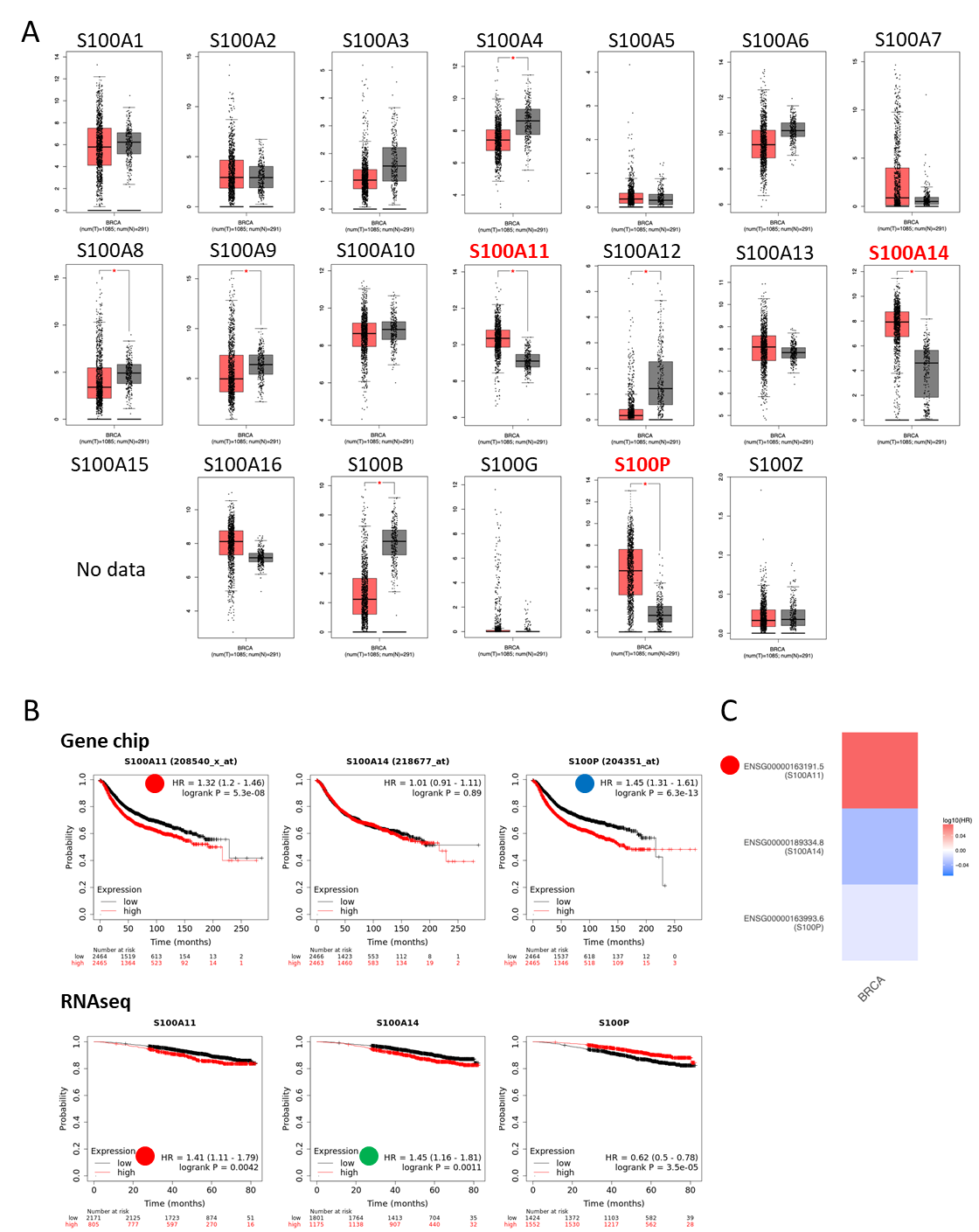
*Supplementary Figure 1.

**SUPPLEMENTARY FIGURE 1. In-silico analysis of S100 genes for their expression and contribution to patient survival. A,** Gene expression plots of S100 family genes from invasive breast carcinoma (BRCA) specimens were obtained from a publicly available website (http://gepia.cancer-pku.cn/). Red: tumor, gray: normal. **B,** Overall survival plots according to S100A11, S100A14, and S100P expression levels in breast cancer patients were obtained from a publicly available website (http://kmplot.com/analysis/). Data are means±SDs. *p<0.05. Breast cancer patients with high expression of S100A11 (red circle) but not S100A14 (green circle) or S100P (blue circle) showed lower survival at significant levels in a constant manner through both the gene chip and RNA-seq data. **C, Among the indicated genes,** high expression of S100A11 (red circle) showed the strongest correlation with lower survival of BRCA patients (GEPIA2: http://gepia2.cancer-pku.cn/#index).

## Supplementary Figure 2.


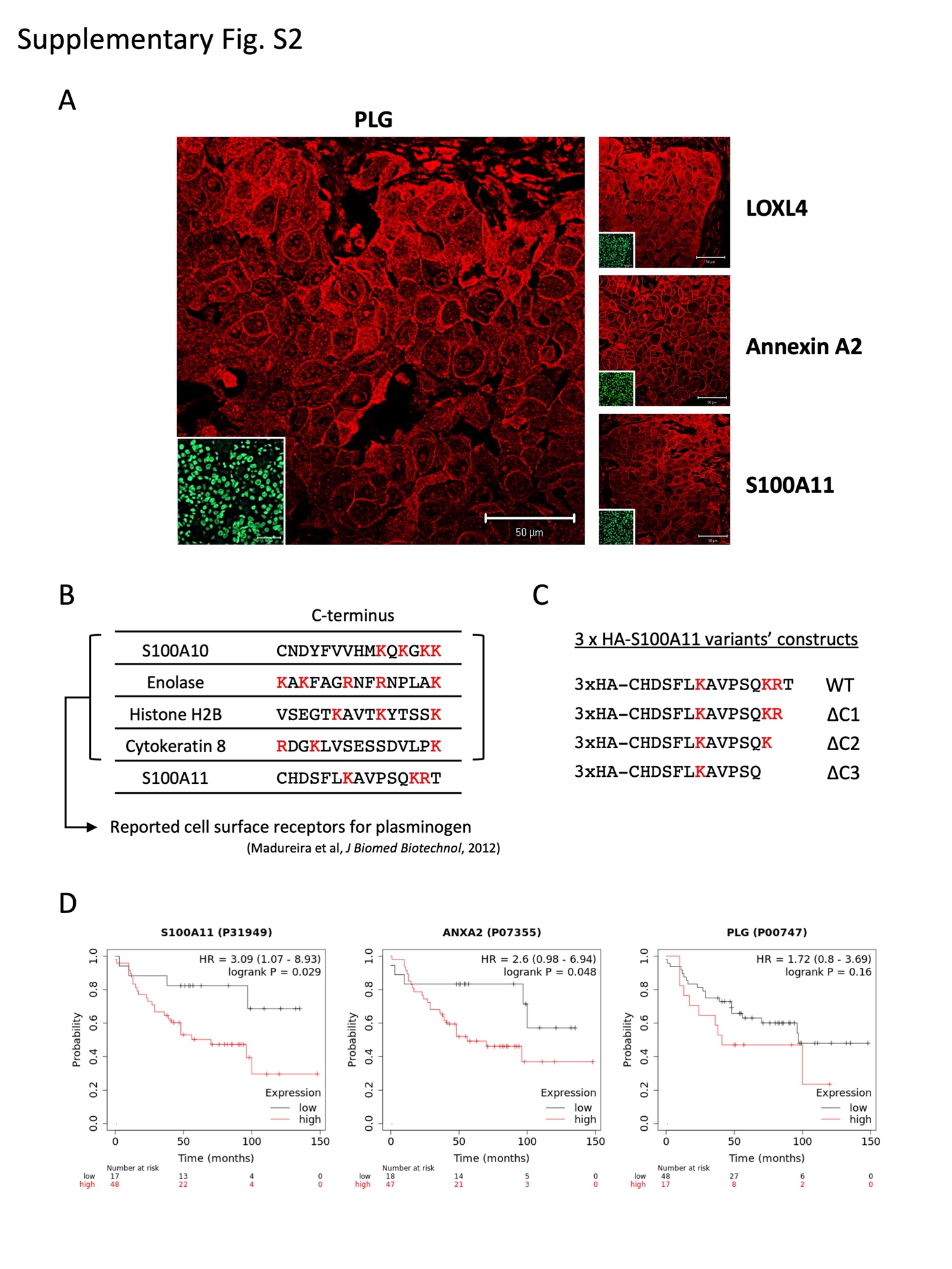


**SUPPLEMENTARY FIGURE 2. Significance of the cell surface annexin A2/S100A11 complex on the capturing of plasminogen and of their constituent protein levels on patient survival. A,** Representative images of immunofluorescence staining were displayed. Plasminogen (PLG), LOXL4, annexin A2, and S100A11 in the breast cancer tissue. Nuclei were stained with SYBR Green. **B,** The C-terminal amino acids elements of the indicated proteins are shown. **C,** The C-terminal deletion constructs of S100A11 were all designed to express as the N-terminal epitope (3 x HA) tagged form. **D,** Overall survival plots according to S100A10, S100A11, annexin A2 (ANXA2), and plasminogen (PLG) protein expression levels in breast cancer patients were obtained from a publicly available website (http://kmplot.com/analysis/).

**
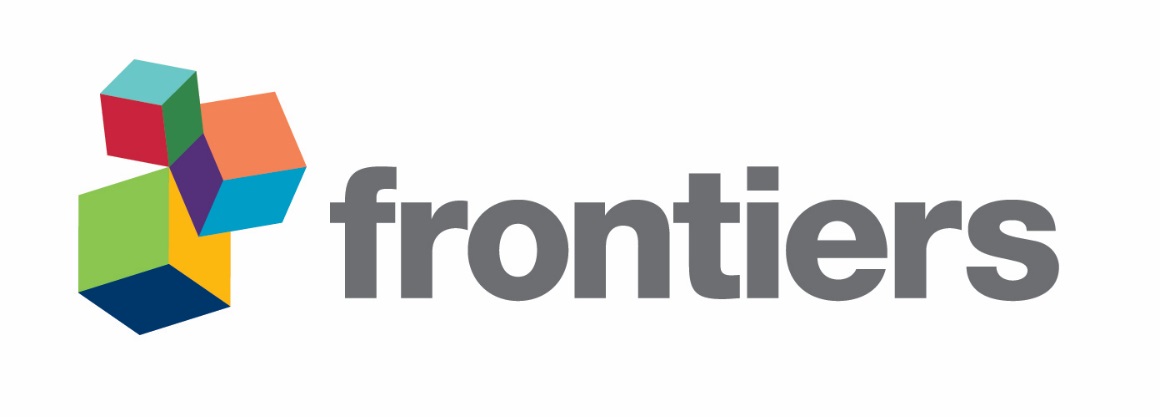
**
